# The role of the social worker in the assessment and management of adults receiving Home Parenteral Nutrition

**DOI:** 10.1038/s41430-024-01555-0

**Published:** 2025-02-03

**Authors:** Sharon Carey, Shannyn Brown, Suzie Ferrie

**Affiliations:** 1 Faculty of Medicine and Health, University of Sydney, Sydney, NSW Australia; 2 Nutrition and Dietetics Department, Royal Prince Alfred Hospital, Sydney, NSW Australia; 3 Institute of Academic Surgery, Royal Prince Alfred Hospital (RPAH), Sydney, NSW Australia; 4 Department of Social Work, Royal Prince Alfred Hospital, Sydney, NSW Australia

**Keywords:** Rehabilitation, Nutrition

## Abstract

People diagnosed with Intestinal Failure (IF) requiring Home Parenteral Nutrition (HPN) have complex medical and nutritional requirements. To date, assessment for, and ongoing management of, HPN has relied on medical, nursing and dietetic input. However, success in assessing suitability for HPN and ongoing management is often reliant on non-medical factors. This paper aims to outline the critical clinical role the social worker can play and their scope of practice in assessing and managing people requiring HPN. This includes assessment across medical, psychosocial, financial, legal, quality-of-life and healthcare-related domains. This perspective paper highlights the vast scope in which the social worker can support people diagnosed with IF requiring HPN. More research is required to quantify the value of the social worker as a key member of the multidisciplinary IF team.

## Introduction

People diagnosed with Intestinal Failure (IF) requiring Home Parenteral Nutrition (HPN) have complex medical, surgical and nutrition management needs [1]. This complexity of illness along with the burden of HPN administration, including associated risks, make this patient group susceptible to reduced Quality of Life (QoL) [2, 3]. Qualitative and quantitative studies have shown that QoL in people living with HPN is impacted by physical (symptoms, complications), psychological (anxiety, depression, negative self-image, low self-confidence), social (social isolation, inability to travel) and financial (loss of income) factors [3–5]. Hence the decision to provide HPN is not only based on the clinical need for HPN, but requires a much more expansive assessment of psychological, social, behavioural and financial factors. The role of supportive care health professionals in assessing patients for, and guiding patients throughout the management of HPN cannot be underestimated. Of particular note is the role of psychology and social work, both of which can guide people in adapting to live with HPN. While there can be overlap between these professions, the psychologist is essential in supporting and providing interventions to ensure the mental well-being of a person living with a chronic illness [6]. Comparatively, the social worker is essential in considering social determinants impacting health and guiding individuals to find solutions including issues related to behaviour and economic difficulties [7]. These health professionals are in a unique position to provide supportive care for people living with HPN and relay invaluable insight into a patient’s situation and capacity back to the multidisciplinary IF team.

The important role of the social worker in HPN assessment and management was outlined in 1981 when this treatment option was first developed [8]. Johnston outlined the importance of the social worker in guiding patients through psychosocial adjustment and social rehabilitation [8]. In the 1980s, the importance of social work in other similar home therapies was also explored including home intravenous antibiotic therapy [9] and home haemodialysis [10]. More recently, Ross et al. emphasised the unique role of social work within the ‘hospital in the home’ service model [7]. However, as the management of IF requiring HPN has evolved, the role of the social worker has been sidelined as services struggle to ensure fundamental medical, nursing and dietetic positions are resourced [11, 12].

Given the recent increased heterogeneity and complexity of people relying on HPN [13] the role of the social worker in this area has expanded greatly over the last 40 years. This paper aims to outline the critical role the social worker can play and their broad scope of practice in assessing and managing people requiring HPN. Table 1 outlines a comprehensive but not definitive checklist of domains (medical, psychosocial, financial, legal, quality-of-life and healthcare engagement) to consider when assessing and managing people with IF requiring HPN.

**Table 1:** Supportive care checklist for assessment and management of people referred for HPN.

Domains	Factors for consideration
Medical	
Current admission	Reason for admission Length of admission Prolonged illness versus sudden illness Level of institutionalisation
Previous medical history	Key health events Ability to adjust during periods of previous illness
Mental health	Current diagnosis and treatments Current and previous psychological support and perceived usefulness Current perception of own mental health Body image and disordered eating
Suicide ideation/attempts	Any current or previous suicide ideation, planning or attempts.
Functionality	Physical (sight, hearing, mobility, dexterity) Cognitive ability Literacy level
Formal support services	Current services and possible need for services Eligibility for services
Compliance with medications	Administration of own medications Forms of medications—tablets, intravenous Ability to adhere to a medication regimen
Family history	Family medical history Personal experiences with past family illness
Psychosocial	
Relationships	Current significant relationships Identification of positive and possibly harmful relationships Current or previous domestic violence
Responsibilities	Parent and carer responsibilities Wider family responsibilities Pets
Community connections	Usual social activities and likely impact of HPN on ability to continue these
Faith and beliefs	Religion and/or faith, and related practices Belief systems impacting engagement with healthcare Health literacy
Support networks	Social supports Use of social media
Sexuality	Sexual orientation Sexual relationships Family planning, fertility and genetic counselling
Substance abuse	Current or previous illicit and prescription drug use Intravenous drug use.
Coping strategies	Locus of control Harmful behaviours to self and others
Financial and legal	
Occupation	Employment to date Education Significance of occupation/employment in individual’s identity and life Enjoyment and fulfilment related to occupation/employment
Incomes and finances	Financial income Key financial costs Private healthcare
Accommodation	Location, type, cost and other occupants at accommodation Suitability for HPN requirements (stairs, flooring, fridge, cleanliness, hygienic space to connect/disconnect HPN)
Legal	Preparation of advanced care planning and will Power of attorney, formal guardianship and/or financial manager provisions Withdrawal of treatment and voluntary-assisted dying
Quality of life	
Future goals	Short, medium and long-term life goals
Expectations of HPN	Understanding of role of HPN in illness management, what it can and cannot do.
Concerns regarding HPN	Understanding complications of HPN Addressing challenges of HPN (e.g. ability to travel)
Healthcare engagement	
Current healthcare involvement	Current and previous healthcare involvement Private healthcare
Relationship with healthcare	Relationship with current and previous healthcare system/provider Previous conflict Perceived trust and communication
Coordination of care	Multidisciplinary referrals and coordinated care Transition of care across services

*HPN* Home Parenteral Nutrition.

### Medical considerations

Due to the heterogeneity of IF, a detailed assessment of the medical and surgical history leading up to HPN assessment will allow substantial insight into the social adjustment challenges each individual may face. People who have experienced a long-standing history of chronic illness (e.g. Crohn’s disease or a persistent dysmotility disorder) may welcome the transition to HPN as a positive step; while those who have experienced a sudden change in health status (e.g. unexpected catastrophic complications following routine surgery) may take much longer, and require more support, for their adjustment to HPN, as well as to their illness and the process of navigating the healthcare system generally [5]. Assessing an individual’s prior experiences (such as any substantial family history of illness and/or HPN), existing relationships with health professionals (including trust and ability to communicate with clinicians) and current adherence to medication regimens will allow an evaluation of likely compliance with the tasks associated with HPN.

Previous or resultant impairments to physical or cognitive function will affect an individual’s ability to administer HPN and their need for additional support. The social worker can often offer a unique insight into the patient’s mindset and aptitude that can be valuable when considering suitability and ability to manage HPN at home. The social worker can play a key role in assessing the adequacy of existing support services, considering whether additional services will be needed for HPN and assisting with applications where required.

A critical aspect of the medical assessment for HPN is to evaluate the individual’s mental health status. Previous and current mental health diagnoses, current treatments and psychological support systems are important to consider. It is highly likely that during times of stress mental health issues may resurface [14] and therefore should be proactively managed when embarking on the process of establishing HPN. Monitoring for signs of distress and suicidal ideation should be ongoing. Formal mental health support systems should be in place where required, ensuring that all members of the healthcare team are aware of the need to monitor for changes in mental health.

### Psychosocial considerations

As the social worker practices under a holistic framework, they often have a unique relationship with each person allowing an exploration of how commencing HPN may affect psychosocial function and vice versa. The social worker will assess the adequacy of social support, including healthy relationships with friends, family and/or carers and assessment of domestic violence risk. The social worker will ensure family and/or carers also have their own support structures in place, and that the carers’ own coping strategies are not harmful to themselves or to the person with IF requiring HPN. Exploring individuals’ habitual coping strategies, including potentially harmful behaviour to self (such as excessive drug use) or to others (such as abuse) is important as requiring HPN and living with a life-threatening chronic illness can impose substantial ongoing stress for many people.

As discussed in 1981, significant social rehabilitation and adaptation is required when commencing HPN [8]. Food is an intrinsic part of many social interactions, and this means that difficulties with food and eating can lead to a reluctance to participate in community events and/or a feeling of social isolation. Consequences of IF requiring HPN can include the inability to consume a wide range of foods, and socially unacceptable gastrointestinal complications (such as uncontrollable flatulence, urgent bowel movements and/or high stoma outputs) which are experienced as embarrassing or stigmatised. The social worker is able to identify these risks and work with the individual to identify strategies to ensure that IF is not an insurmountable barrier to enjoyable social functioning.

In recent years, the use of social media has become more common which allows people with rare diseases to connect with one another [15]. This can reduce feelings of isolation, however, it is important to assess the level of engagement with social media and the possible positive or negative impact of social media for each individual [16]. Importantly, education needs to emphasise that each person with IF requiring HPN will have individualised medical care requirements and needs to understand that lay opinions related to medical care are not always based on evidence and can be potentially harmful.

An individual’s health beliefs and health literacy can have a significant impact on their ability to adhere to the recommended HPN regimen and other aspects of their medical care. The social worker can be very helpful in elucidating the role of an individual’s faith and belief systems related to health and medicine, including special food requirements, ritual periods of fasting, and the individual’s understanding of the requirements of their therapeutic management and how this is prioritised within other aspects of their life.

Dependent on age and life stage, concerns around sexuality and sexual relationships may be relevant, particularly with consideration to night-time HPN regimens, stomas, fistulas, drains and feeding tubes. Body image, self-identity and social relationships can be profoundly affected by these changes when an individual starts on HPN. The social worker can have a valuable role in exploring these issues, as well as facilitating discussions around family planning, fertility treatments and possible need for genetic counselling if required.

### Financial and legal considerations

Financial burden is common in people living with chronic illness [17] although this varies widely between different countries and their healthcare systems and health funding arrangements. There is minimal information documented on the financial impact of IF requiring HPN from the patient's perspective [18]. An assessment of current and ongoing financial status, need for financial support, and eligibility for social services, is essential to minimise financial stress. Due to the life-threatening nature of IF and HPN, people may wish to address issues around advanced care planning and wills, power of attorney, and formal guardianship and/or financial manager arrangements. As this cohort of people living with IF requiring HPN approach older age, issues around withdrawal of treatment and voluntary-assisted dying will become more common. The social worker’s role in supporting the individual in these areas is essential. Advocacy for appropriate housing may also be required where current housing is not suitable. The physical assessment of accommodation regarding suitability for HPN may require referral to an Occupational Therapist. However, this assessment of the physical space needs to be considered within the context of affordability of any modifications, and the impact on other occupants.

### Quality of life considerations

Given the restrictions HPN places on individuals and their families/carers, it is important to assess the expectations that individuals (and their families) have in relation to their life with HPN. Understanding the person’s social context, lifestyle and preferred activities prior to illness will provide insight into the adjustments that will be required when on HPN and how substantial those adjustments will be. Open discussions with individuals on how they plan to manage these adjustments and brainstorming management strategies can relieve future stress and prevent disappointment. The social worker can help with the process of managing the individual’s expectations, particularly to ensure that they have a clear understanding of the role of HPN in the management of their illness, and also of the risks associated with HPN. The aim of HPN is to address nutrition and hydration-related imbalances [19]. Many of the symptoms of IF are unrelated to these imbalances, and thus will still persist when the person is on HPN; the HPN is not a solution for all illnesses related to IF.

### Healthcare engagement

People with IF requiring assessment for HPN have complex health needs often requiring referral to multiple specialist teams. The social worker can identify the need for and initiate many of these referrals. Their skill set is ideal for providing coordination of care to bring teams together to ensure the individual and their family/carers are supported to make informed decisions.

A particular challenge that is becoming more common is the transition of people with IF requiring HPN from paediatric into adult services. While there is a lack of research exploring the needs of people living with IF requiring HPN transitioning from paediatric to adult services, research in other chronic illness patient groups indicates this period of transition can be a confusing and challenging time [20]. If not coordinated well, this transition can leave the young adult disengaged or feeling abandoned as they have left the paediatric service but clinician–patient relationships with the adult service have not fully developed [20, 21]. The social worker can support the delicate transition for both the young adult and their parent/carer, and the gradual shifting of responsibility from parent/carer to self-management [22].

## Conclusion

During the process of assessing a person with IF as to whether they require and are suitable for HPN, there is a large focus on the medical, surgical, and nutritional aspects of care. With limited resourcing, behavioural, psychological and social assessments are often not available. However, the scope and complexity of non-medical factors impacting delivery of medical care is vast. This paper has highlighted some key supportive care factors but is in no way complete. Assessment taking these factors into consideration can mean the difference between successful and unsuccessful use of HPN therapy, and subsequent impact to QoL. There is a dearth of literature around the role of supportive care services in the management of people living with IF requiring HPN, and specifically a lack of interventions showing the impact of such services. This lack of evidence makes it difficult for IF services to develop a strong argument for the inclusion of a social worker within the multidisciplinary team. Nevertheless, the social worker can be an invaluable member of the IF team to ensure patients are supported during assessment and management of HPN.
